# Nanobody-Directed Specific Degradation of Proteins by the 26S-Proteasome in Plants

**DOI:** 10.3389/fpls.2018.00130

**Published:** 2018-02-09

**Authors:** Bianca Baudisch, Ingrid Pfort, Eberhard Sorge, Udo Conrad

**Affiliations:** Phytoantibody Group, Department of Molecular Genetics, Leibniz Institute of Plant Genetics and Crop Plant Research, Gatersleben, Germany

**Keywords:** protein degradation, GFP, transgenic plants, proteasome, deGradFP

## Abstract

Here, we present data showing the directed degradation of target proteins recognized by a specific nanobody in transgenic plants. Green fluorescent protein was depleted by a chimeric nanobody fused to a distinct F-box domain, which enables protein degradation *via* the ubiquitin proteasome pathway. This technique could thus be used to knock out other proteins of interest *in planta* using specific, high-affinity binding proteins.

## Introduction

**FIGURE 1:**
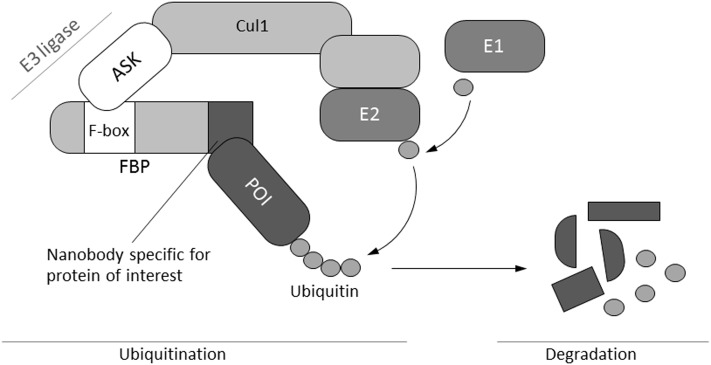
Schematic illustration of the mechanism of selective protein degradation. Protein degradation by the ubiquitin pathway is carried out by a complex cascade of enzymes (E1–E3) that catalyze the covalent attachment of multiple ubiquitin molecules to the target protein. Subsequently, polyubiquitinated proteins are degraded by the proteasome. The N-terminal F-box domain typically binds to one of the members of the ASK family, whereas the C-terminal part determines substrate specificity *via* different protein–protein interaction motifs. These motifs are replaced by a nanobody specific for a protein of interest in order to engineer a molecular tool for selective protein depletion (Caussinus et al., 2011). Cul1, cullin; FBP, F-box protein; ASK, *Arabidopsis*-S-phase kinase-associated protein (SKP1)-like; E1, ubiquitin activating enzyme; E2, ubiquitin conjugating enzyme; E3-ligase, ubiquitin ligase; POI, protein of interest.

Modern cell biology approaches require an understanding of biomolecular pathways and extensive knowledge of protein–protein interactions as a basis of regulatory networks. The manipulation of regulatory and coding sequences by classical mutagenesis, by CRISPR/Cas9 knockout (Bortesi and Fischer, 2015) or by targeting specific transcripts with RNAi, is widely used for analyzing protein function in detail. Whereas genome-wide comparative analysis of transcript and protein levels in *Arabidopsis* suggests that regulation primarily occurs at the transcript level, in many cases, transcript abundances do not necessarily reflect the abundances of the corresponding proteins (Baerenfaller et al., 2008). Direct influence at the protein level could be a more effective way to study protein functions in plants *in vivo*. Immunomodulation of regulatory compounds by the expression of specific recombinant antibodies has been developed as a tool to directly affect the function of desired targets in plant cells. The blockage or change of phytohormone functions *via* plantibodies by building artificial sinks (Artsaenko et al., 1995) or by direct interaction (ten Hoopen et al., 2007) has been reported in several papers (Conrad and Manteuffel, 2001). Functional plant protein (Miroshnichenko et al., 2005) and plant viral protein (Tavladoraki et al., 1993; Boonrod et al., 2004) blockage by *in planta* expression of recombinant antibodies has also been described. The selection of antibodies that specifically inhibit protein functions could be a rather difficult task. The specific intracellular degradation of functional proteins would be a useful solution to this problem. In animal and yeast cells, the ubiquitin-proteolytic apparatus (Ravid and Hochstrasser, 2008) has been manipulated by altering the substrate recognition domain of ubiquitin-protein ligases. Chimeric substrate receptors or peptide–small molecule hybrids could cause the intended target to interact with the substrate receptor of the E3-ligases to allow for the directed degradation of selected proteins (Zhou et al., 2000; Sakamoto et al., 2001; Zhou, 2005). In a similar approach, researchers have adapted an auxin-dependent protein degradation pathway that enables plants to degrade auxin transcription repressors (AUX/IAA) by a specific S-phase kinase-associated protein 1 (SKP1), cullin (CUL1), and F-box protein-containing complex (SCF)–ubiquitin E3-ligase complex. This system allows for a rapid and inducible depletion of target proteins in a reversible and tunable manner by the phytohormone auxin in budding yeast and several animal cells, including human cells, but not in plant cells (Nishimura et al., 2009). New regulation principles introduced by this technology fit into synthetic biology approaches. Caussinus et al. (2011) developed a method for specific protein degradation by replacing the target recognition sequence of the F-box protein by a target-specific nanobody in *Drosophila* and human cells. The degradation of nuclear proteins fused to green fluorescent protein (GFP) using anti-GFP nanobody-targeted E3-ubiquitin ligase complexes in mammalian cells and zebrafish embryos has also been shown (Ju Shin et al., 2015). Nanobodies are stable, small, single-domain antibodies that can be selected by phage display (Muyldermans, 2013). In principle, a ubiquitous approach is possible because specific nanobodies for virtually any protein can be selected with this method. Off-target effects could be avoided by performing extensive selection and characterization of nanobodies with specific binding parameters. To specifically degrade GFP, we expressed the fusion protein NSlmb-VHHGFP4 in the cytosol of transgenic plant cells that showed overexpression of GFP (**Figure 1**). Experimental analysis of the leaf material showed evidence of GFP depletion. With this experiment, we show for the first time that nanobody-driven directed degradation of proteins can also be used in plants. This allows for a plethora of experiments to analyze the role of single regulatory proteins and adds an important component to an integrated synthetic biology concept for plants.

## Materials and Methods

### Construction of Expression Vectors

Cloning was performed as previously described (Sambrook, 2001). DreamTaq polymerase, FastDigest restriction enzymes, and T4 DNA ligase from Thermo Fisher Scientific were used according to the manufacturer’s instructions.

### Construction of the GFP Plant Expression Vector

A *Xba*I/*Xho*I fragment containing the CaMV-enhanced 35S promoter (d35S) was released from plasmid d35S-Nos-AB-M (Himmelbach et al., 2007) and inserted in the pGFP-Amp vector to form pGH102. The *Sfi*I fragment of vector pGH102 harboring the full GFP expression cassette was introduced into the appropriate sites of plasmid p6d35S (Hensel et al., 2015) to generate the vector pGH219.

### Construction of NSlmb-VHHGFP4 and NSnoFbox-VHHGFP4 Plant Expression Vectors

Template sequences (NSlmb-VHHGFP4 and NSnoFbox-VHHGFP4) were provided by Caussinus et al. (2011). NSlmb-VHHGFP4 comprises the sequence encoding the F-box domain containing in the N-terminal part of the F-box protein supernumerary limbs (Slmb) from *Drosophila melanogaster* fused to the GFP-binding nanobody VHHGFP4 sequence. NSnoFbox-VHHGFP4 lacks the F-box domain for SKP1 binding and represents the negative control used in later GFP-depletion assays. The primer pair Slmb-NcoI-for and Slmb-NotI-rev was used to add *Nco*I and *Not*I sites by PCR. The resulting fragments were subcloned using the TOPO Cloning Kit (Thermo Fisher Scientific). After the plasmids were sequenced and cut with *Nco*I and *Not*I, the fragment was inserted into the pRTRA vector. The resulting expression cassette was cut using *Hind*III and ligated into the binary vector pCB301-Kan. The resulting plasmids (containing the NSlmb-VHH-GFP4 or NSnoFbox-VHHGFP4 constructs) were used for transformation of *Agrobacterium tumefaciens* strain GV2260.

### Production of Transgenic Plants

Transformation of *Nicotiana tabacum* was performed by agroinfection of leaf discs (Horsch, 1985) as described by Floss et al. (2010). The leaf discs were submerged for 1 h in an *A. tumefaciens* culture, plated on MS medium, and stored at 24°C in the dark for 2 days. Then, the explants were transferred to NBKC medium (MS medium containing 0.2 mg/L α-naphthaleneacetic acid, 1 mg/L 6-benzylaminopurine, 50 mg/L kanamycin, and 500 mg/L cefotaxime). Every 10–14 days, the plant material was removed and placed in fresh NBKC medium until plantlets appeared. Plantlets (2–3 cm in height) were placed onto MS medium containing 50 mg/L kanamycin for selection. Surviving putative transgenic plants were planted in soil and grown to maturity in a greenhouse.

### SDS-PAGE and Western Blotting

Leaf discs were stored at -80°C, transferred to 2 mL safe-lock tubes along with two metal bullets, and disintegrated in a Retsch mill at a frequency of 28/s for 2 min under liquid nitrogen. Then, 150 μL of 2× SDS sample buffer (Conrad et al., 1998) was added and heated at 95°C for 10 min. Samples were then cooled on ice and centrifuged for 30 min at 21,000 × g. Extracted plant proteins (corresponding to 40 μg protein) were separated by reducing SDS–PAGE (12% polyacrylamide) and then electrotransferred to nitrocellulose membranes. The Western blotting procedure was carried out using monoclonal anti-cmyc antibodies following the protocol described by Gahrtz and Conrad (2009). The secondary antibody was sheep anti-mouse IgG horseradish-peroxidase-linked whole antibody (GE Healthcare UK Ltd., Little Chalfont, Buckinghamshire, United Kingdom), and ECL was used for detection. GFP was detected with specific rabbit anti-GFP antibodies and goat anti-rabbit IgG horseradish-peroxidase-linked whole antibody. Specific signals were detected as described above.

### Native Extraction of GFP from Tobacco Leaves

Around 100 mg of tobacco leaves was transferred to 2 mL safe-lock tubes along with two metal bullets and disintegrated in a Retsch mill at a frequency of 28/s for 2 min under liquid nitrogen. Then, 300 μL of extraction buffer [50 mM Tris, pH 8.0, 5 mM EDTA, 1 mM DDT, 1× Complete proteinase inhibitor mix (Roche, Mannheim, Germany)] was added and the extracts were shaken end-over-end for 45 min at 4°C. To analyze the stability in solution, 50 μM MG-132 (Sigma, Steinheim, Germany) was applied.

### Competitive ELISA to Measure GFP

Enhanced green fluorescent protein (kindly provided by Mario Jakob, Universität Halle/Saale, Germany) was diluted in Phage PBS (100 mM NaCl, 32 mM Na_2_HPO_4_, 17 mM NaH_2_PO_4_, pH 7.2) at a concentration of 0.05 μg/100 μL and put into Immunoplate MaxiSorp wells (Nalge Nunc International, Roskilde, Denmark). After overnight incubation at room temperature, the wells were saturated with 3% bovine serum albumin (BSA) in phosphate buffered saline, 0.05% Tween 20 (PBS-T) (Gahrtz and Conrad, 2009). Standards (0.01–100 nM eGFP) and appropriately diluted native extracts were mixed with rabbit anti-GFP antibodies (diluted 1:50,000 in 3% BSA–PBS-T) and incubated at 25°C for 30 min in a master plate. Samples were then transferred to the saturated eGFP plates and incubated for 1 h at 25°C. After extensive washing with PBS-T, goat anti-rabbit IgG alkaline-phosphatase-linked whole antibody (Sigma, Steinheim, Germany), diluted 1:2000 in 3% BSA and PBS-T, was applied for 1 h at 25°C. After further washing the enzymatic substrate, *p*-nitrophenyl phosphate (pNPP) in 0.1 M diethanolamine-HCl (pH 9.8) was added, and the absorbance signal was measured at 405 nm after 1 h incubation at 37°C. Measured values from control experiments performed in parallel (same handling procedure but without the antigen incubation step) were subtracted.

### Detection of Gene Expression by RT-PCR

The OneTaq One-Step RT-PCR Kit (New England Biolabs) was used to identify GFP and actin mRNA. Analysis was performed according to the manufacturer’s instructions. Briefly, total RNA from plant tissue was extracted using the RNeasy Plant Kit (Qiagen). For cDNA synthesis the following solutions were mixed and denatured at 70°C for 5 min: 10 μL precipitated RNA (1 μg), 9 μL of H_2_O, 2 μL of 10 μM GFP reverse primer or 2 μL of 10 μM actin reverse primer, and 25 μL of OneTaq One-Step Quick-Load Reaction Mix. Then, 2 μL of 10 μM GFP forward primer or 10 μM actin forward primer and 1.8 μL of OneTaq One-Step Enzyme Mix were added. PCR amplification was performed according to the following protocol for 40 cycles: 48°C 15 min, 94°C 1 min, 94°C 15 s, 63°C 30 s, 58°C 1 min, and 68°C 5 min. The resulting DNA fragments were separated on a 2% agarose gel in tris-acetate-EDTA, pH 8.0 (TAE) buffer (Sambrook, 2001).

### Primer Sequences

ASK9/10-BamHI-for 5′-ATGGATCCTCGACGAAGAAGATCATA-3′

ASK9-XhoI-rev 5′-TTCTCGAGTTCAAAAGCCCATTTATTCTC-3′

ASK10-XhoI-rev 5′-TTCTCGAGTTCAAAACCCCATTGATTCT-3′

Slmb-NcoI-for3 5′-CCATGGCCATGATGAAAATGGAGACTGA-3′

Slmb-NotI-rev5 5′-TTGCGGCCGCGCTGGAGACGGTGACCTG-3′

Slmb-BamHI-for3 5′-GCGGATCCATGATGAAAATGGAGACTGAC-3′

Slmb-NotI-rev5 5′-TTGCGGCCGCGCTGGAGACGGTGACCTG-3′

Slmb-NdeI-for3 5′-CATATGATGATGAAAATGGAGACTGACAAAAT

AATGGACGAAACCAACTCCAATGCACAGGCC-3′

Slmb-cmyc-NotI-rev5 5′-GCGGCCGCATTCAGATCCTCTTCTGAGATGAG

 TTTTTGTTCGTCGACGCTGGAGACGGTGACCTG-3′

VHHGFP4-NdeI-for 5′-GCATATGGATCAAGTCCAACTGGTGGAGT-3′

VHHGFP4-SalI-rev 5′-GTCGACGCTGGAGACGGTGACCTG-3′

BamHI-VHHGFP4-for 5′-GGATCCATGGATCAAGTCCAACTGGTG-3′

NotI-VHHGFP4-rev 5′-GCGGCCGCGCTGGAGACGGTGA-3′

Actin forward 5′-CTATTCTCCGCTTTGGACTTGGCA-3′

Actin reversed 5′-AGGACCTCAGGACAACGGAAACG-3′

GFP forward 5′-ATGGTGAGCAAGGGCGAGGAGCT-3′

GFP reversed 5′-TTACTTGTACAGCTCGTCCATGCCGA-3′

## Results

### Design of Expression Vectors and Production of Transgenic Plants Expressing NSlmb-VHHGFP4, NSnoFbox-VHHGFP4, and GFP

**FIGURE 2:**
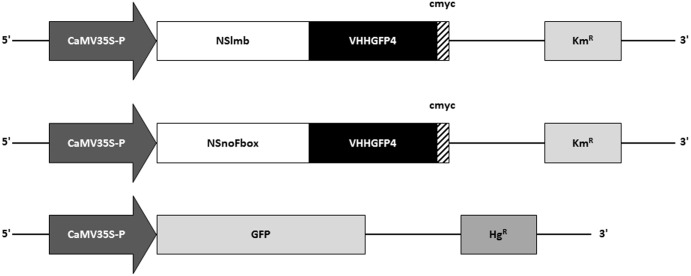
Schematic depiction of constructs used for expression of NSlmb-VHHGFP4, NSnoFbox-VHH, and GFP in transgenic tobacco plants. NSlmb-VHHGFP4 and NSnoFbox-VHH transgenic plants (Km^R^) have been overtransformed with the GFP expression construct (Hg^R^). Km^R^, kanamycin resistance; Hg^R^, hygromycin resistance; CaMV35S-P, CaMV35S ubiquitous promoter; NSlmb, F-box protein from *Drosophila* (Caussinus et al., 2011); NSnoFbox, NSlmb with deleted F-box domain (Caussinus et al., 2011); GFP, green fluorescent protein; VHH, nanobody; cmyc, cmyc polypeptide protein tag.

**FIGURE 3:**
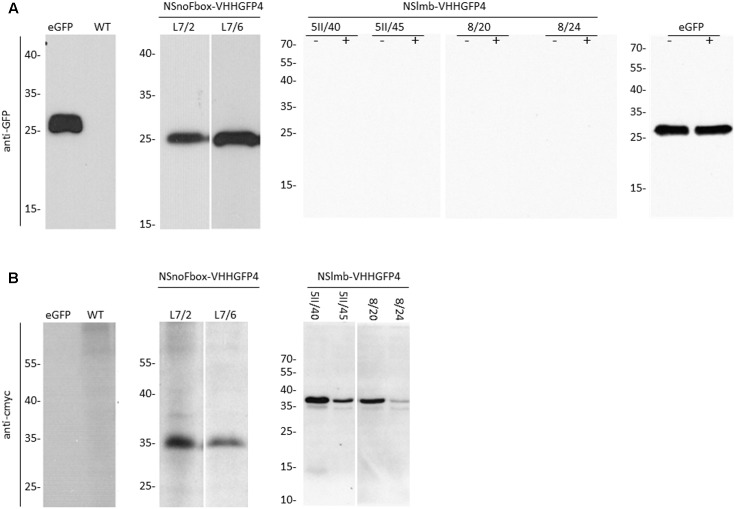
Protein expression analysis (Western blot) with anti-GFP antibodies, anti-rabbit peroxidase, and ECL **(A)** and anti-cmyc antibodies, anti-mouse peroxidase, and ECL **(B)**, of transgenic lines expressing NSlmb-VHHGFP4 and GFP (5II/40, 5II/45, 8/20, 8/24), NSnoFbox-VHHGFP4 and GFP (L7/2, L7/6), and GFP (GFP) and of tobacco wild-type (WT) plants. NSnoFbox-VHHGFP4 (fusion protein with deleted F-box domain) and WT plants served as controls. The size of molecular marker proteins is given in kilodalton. The expected molecular weights of GFP, NSlmb-VHHGFP4, and NSnoFbox-VHHGFP4 are 27, 39, and 35 kDa, respectively. Extracts of 5II/40, 5II/45, 8/20, 8/24, and GFP have been treated with MG-132 (+) or not (–). In both cases GFP protein expression is lacking in 5II/40, 5II/45, 8/20, and 8/24. eGFP, pure eGFP.

We constructed expression vectors allowing for the ubiquitous expression of either an NSlmb-VHHGFP4 (anti-GFP nanobody) or an NSnoFbox-VHHGFP4 fusion protein in the cytosol of plant cells. The CaMV35S promoter induces expression in nearly all plant cells, and the cmyc Tag enables detection of the fusion proteins by Western blot analysis (**Figure 2**). All expression cassettes were cloned into a binary plant expression vector allowing for the selection of transgenic plants *via* kanamycin resistance (Km*^R^*). The second construct served as an internal control to show that the degradation of GFP was F-box domain dependent. We decided to use an animal-derived F-box variant and not a plant F-box variant to minimize the effects on general regulatory processes in plants. After leaf-disc transformation experiments were completed, 51 kanamycin-resistant plants transformed with NSlmb-VHHGFP4 were selected, grown, and analyzed by Western blot. Twenty lines with transgenic protein accumulation were detected. In addition, 51 kanamycin-resistant plants transformed with NSlmb-VHHGFP4 were selected, grown, and analyzed by Western blot. Seven lines with transgenic protein accumulation were detected. A binary plant expression vector (pGH219) allowing for the ubiquitous cytosolic expression of GFP, also driven by the CaMV35S promoter, was used for super-transformation of NSlmb-VHHGFP4 and NSnoFbox-VHHGFP4 lines. This vector allows for the selection of hygromycin-resistant transgenic plants (**Figure 2**). Wild-type *N. tabacum* (tobacco) plants were transformed as controls and GFP expression was detected by rabbit anti-GFP antibodies (**Figure 3**).

### Directed Degradation of GFP in Transgenic Tobacco Plants

**FIGURE 4:**
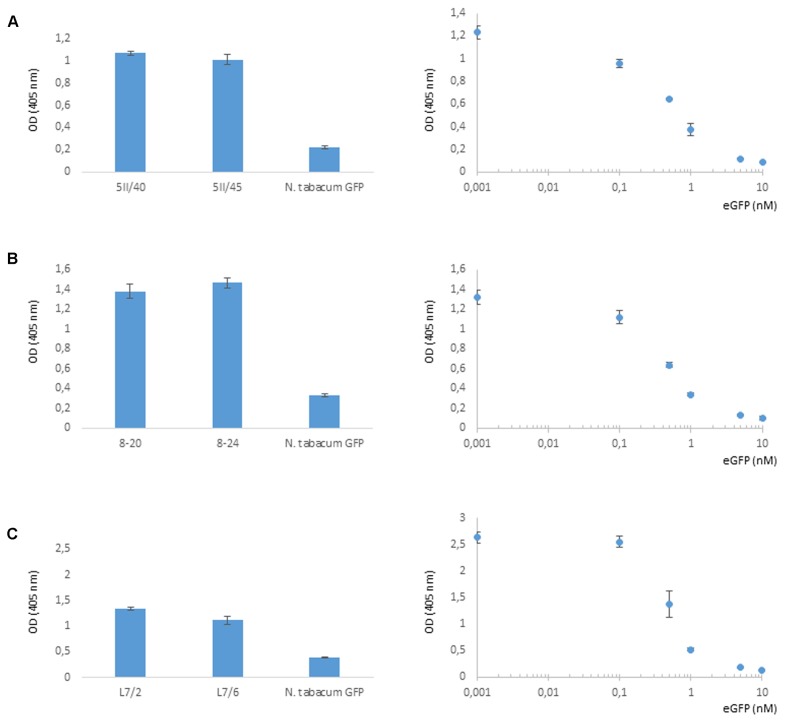
Selective degradation of GFP driven by NSlmb-VHHGFP4 fusion proteins *in planta* measured by competitive ELISA for quantification of GFP. Five parallels of each native plant extract dilution as well as five parallels of each eGFP standard dilution were incubated and measured on the same ELISA plate. GFP plant extracts (*N. tabacum* GFP) were also measured on each plate. Resulting arithmetic means were presented including standard deviations given as bars. **(A,B)** Transgenic lines expressing NSlmb-VHHGFP4 and GFP. **(C)** Transgenic lines expressing NSnoFbox-VHHGFP4 and GFP.

**FIGURE 5:**
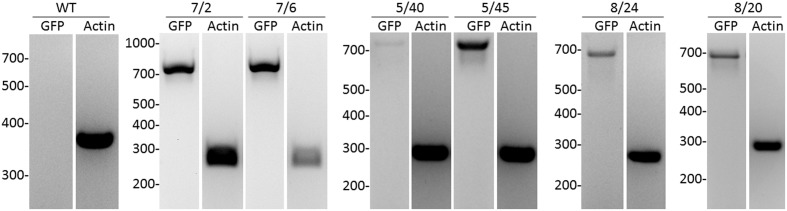
Analysis of GFP transcripts in transgenic tobacco plants by reverse transcription and PCR compared to actin transcription. As internal standard, a housekeeping gene encoding for ribosomal protein actin9 in *N. tabacum* was analyzed (Volkov et al., 2003). NSlmb-VHHGFP4 (8/20, 8/24, 5/40, 5/45), NSnoFbox-VHHGFP4 (L7/2, L7/6), and wild-type plant (WT) were analyzed. The expected length/size of the PCR products was 720 bp for GFP and 260 bp for actin.

Four independent lines expressing the NSlmb-VHHGFP4 fusion protein and two independent lines expressing the NSnoFbox-VHHGFP4 fusion protein were super-transformed with a transgene encoding GFP as a reporter for protein degradation. Overall, 11 kanamycin- and hygromycin-resistant tobacco lines deriving from different super-transformation experiments were selected that showed NSlmb-VHHGFP4 accumulation, but little to no GFP accumulation was observed by Western blot analysis using highly specific rabbit anti-GFP antibodies (**Figure 3** and **Table 1**). Conversely, strong GFP accumulation was detected in transgenic plants expressing NSnoFbox-VHHGFP4. Six kanamycin/hygromycin lines (derived from two NSnoFbox-VHHGFP4 fusion protein expressing lines) were analyzed that showed NSnoFbox-VHHGFP4 fusion protein expression and GFP expression. The results of the analysis of two NSnoFbox-VHHGFP4-GFP lines are demonstrated in **Figure 3**. To strengthen these results quantitatively, GFP accumulation was measured by a competitive ELISA, which could detect GFP concentrations between 4 and 0.1 nM (**Figure 4**). In all nine F-box transgenic plants investigated by the competitive ELISA, GFP concentrations were determined to be below 0.1 nM (detection limit, **Figure 4** and **Table 1**). GFP concentration was significantly higher in NSnoFbox-VHHGFP4-GFP lines and in GFP expressing tobacco plants (**Figure 4**). In further experiments, we wanted to rule out that the lack of GFP protein expression was due to loss of the transgene or to downregulation at the transcriptional level. Using reverse transcription and PCR amplification with specific primers, we showed that NSlmb-VHHGFP4 plants contained GFP transcripts in similar amounts to NSnoFbox-VHHGFP4 plants (**Figure 5**). The detection of transcripts indicates the presence of the transgene coding for GFP. Consequently, the lack of GFP accumulation in NSlmb-VHHGFP4 plants detectable *via* Western blot analysis is not due to downregulation at the transcriptional level. To rule out, that the removal of GFP by directed proteolysis did occur after extraction, we added MG132, a potent inhibitor of the proteasome (Lee and Goldberg, 1998) to the extraction buffer. The samples, that have been either treated with MG132 or not, show identical bands for the NSlmb-VHHGFP4 plants, the NSnoFbox-VHHGFP4 plants, and the GFP plants, as well (**Figure 3**). These results clearly demonstrate that GFP is degraded at the protein level in the presence of the F-box domain in the plant cells, whereas the lack of the F-box domain leads to GFP accumulation (summarized in **Table 1**).

**Table 1:** Summary of anti-GFPVHH F-box fusion protein accumulation measured by anti-cmyc Western blot, GFP protein accumulation measured by anti-GFP Western blot, and competitive ELISA and GFP transcript expression detected by reverse transcription and PCR.

No.	Anti-cmyc Western	Anti-GFP Western	Anti-GFP ELISA	GFP transcript
5I/5	+	-	Not done	+
5I/88	+	-	Not done	+
5II/21	+	- (+)	<0.1 nM	+
5II/22	+	-	<0.1 nM	+
5II/24	+	- (+)	<0.1 nM	+
5II/31	+	- (+)	<0.1 nM	+
5II/40	+	-	<0.1 nM	+
5II/45	+	-	<0.1 nM	+
8/15	+	-	<0.1 nM	+
8/20	+	-	<0.1 nM	+
8/24	+	-	<0.1 nM	+


### *Drosophila* F-Box Domain Can Induce Selective Protein Degradation in Plant Cells

The interaction of the F-box domain with plant SKP1 (Vierstra, 2009) is presumed to have caused the effect described above. SKP1-like proteins, called ASKs, have been described in plants (Zhao et al., 2003). The specific interaction between the F-box domain of *Drosophila* and SKP1-like proteins in plants that act as adaptors to link the F-box protein to the scaffold of the SCF complex (**Figure 1**) has been shown by *in vitro* ELISA interaction studies (see Supplementary Material). This result shows that the F-box domain from *Drosophila* is capable of recognizing the SKP1-analogous ASK proteins of plants.

## Discussion

The 26S proteasome, a ubiquitous machine in eukaryotes, could be used to manipulate protein functions *in vivo.* Manipulation, in this sense, means degradation of the functional protein of interest. This would aid in learning more about specific regulatory processes or designing new pathways in the context of new concepts in plant breeding and secondary metabolite engineering (Staniek et al., 2013). We show in the present paper that the degradation of solitary GFP molecules was possible in plants. This is a crucial first step toward directly targeting plant proteins and removing them to gain new insights into their functions. Interestingly, in *Drosophila*, the modified SCF E3-ligase could not process solitary GFP molecules, whereas GFP fusion proteins were degraded (Caussinus et al., 2011). A major benefit of this new technology is strong and selective degradation. Off-target effects could only be caused by nonspecific antibody binding. Here, several types of small, specific binders have recently been developed that all are selectable and allow specific and high-affinity binding. This includes single-chain Fv (scFv) (Bird et al., 1988) and nanobodies (Muyldermans, 2013), the most common forms of recombinant antibodies. The direct expression of scFv in the cytoplasm of target cells leads in only a few cases (<1%) to sufficient stable intrabodies (Auf der Maur et al., 2004; Visintin et al., 2004). Binders with high-affinity constants could be enriched by mimicking affinity maturation *in vitro* with phage display (Chowdhury and Pastan, 1999). Nanobodies are beneficial in this context, because they have a low molecular weight and a stable immunoglobulin antigen binding unit and can recognize cryptic epitopes (Helma et al., 2015). Synthetic biology uses recombinant binders to control gene transcription, to change signaling cascades and to influence protein turnover (Lienert et al., 2014). In general, a central idea of the synthetic biology approach is the rational design of genetic building blocks. Novel cellular functions could be created and designed that target several new purposes and applications (Helma et al., 2015). In this paper, we show the targeted proteasomal knockdown of GFP by use of a specific anti-GFP nanobody in plants. Engineering of plants in terms of resistance development by removing pathogen effector proteins is one potential application of targeted protein degradation. This concept of selective protein degradation, now proven in principle, could also be used to manipulate regulatory pathways in plants. A plethora of experiments using selected degradation and selected activation of genes by specific nanobodies could be performed and lead to developing new strategies in crop plant design for agriculture in the future.

## Author Contributions

BB and UC planned and designed the research. BB, IP, and ES performed the experiments and analyzed the data. UC and ES wrote the manuscript.

## Conflict of Interest Statement

The authors declare that the research was conducted in the absence of any commercial or financial relationships that could be construed as a potential conflict of interest.

## References

[B1] Artsaenko O. Peisker M. zur Nieden U. Fiedler U. Weiler E. W. Muntz K. (1995). Expression of a single-chain Fv antibody against abscisic acid creates a wilty phenotype in transgenic tobacco. *Plant J.* 8 745–750. 10.1046/j.1365-313X.1995.08050745.x 8528285

[B2] Auf der Maur A. Tissot K. Barberis A. (2004). Antigen-independent selection of intracellular stable antibody frameworks. *Methods* 34 215–224. 10.1016/j.ymeth.2004.04.004 15312674

[B3] Baerenfaller K. Grossmann J. Grobei M. A. Hull R. Hirsch-Hoffmann M. Yalovsky S. (2008). Genome-scale proteomics reveals *Arabidopsis thaliana* gene models and proteome dynamics. *Science* 320 938–941. 10.1126/science.1157956 18436743

[B4] Bird R. E. Hardman K. D. Jacobson J. W. Johnson S. Kaufman B. M. Lee S. M. (1988). Single-chain antigen-binding proteins. *Science* 242 423–426. 10.1126/science.3140379 3140379

[B5] Boonrod K. Galetzka D. Nagy P. D. Conrad U. Krczal G. (2004). Single-chain antibodies against a plant viral RNA-dependent RNA polymerase confer virus resistance. *Nat. Biotechnol.* 22 856–862. 10.1038/nbt983 15195103

[B6] Bortesi L. Fischer R. (2015). The CRISPR/Cas9 system for plant genome editing and beyond. *Biotechnol. Adv.* 33 41–52. 10.1016/j.biotechadv.2014.12.006 25536441

[B7] Caussinus E. Kanca O. Affolter M. (2011). Fluorescent fusion protein knockout mediated by anti-GFP nanobody. *Nat. Struct. Mol. Biol.* 19 117–121. 10.1038/nsmb.2180 22157958

[B8] Chowdhury P. S. Pastan I. (1999). Improving antibody affinity by mimicking somatic hypermutation in vitro. *Nat. Biotechnol.* 17 568–572. 10.1038/9872 10385321

[B9] Conrad U. Fiedler U. Artsaenko O. Phillips J. (1998). “Single-chain Fv antibodies expressed in plants,” in *Recombinant Proteins from Plants: Production and Isolation of Clinically Useful Compounds*, eds Cunningham C. Porter A. J. R. (Totowa, NJ: Humana Press), 103–127. 10.1007/978-1-60327-260-5_9

[B10] Conrad U. Manteuffel R. (2001). Immunomodulation of phytohormones and functional proteins in plant cells. *Trends Plant Sci.* 6 399–402. 10.1016/S1360-1385(01)02043-X 11544111

[B11] Floss D. M. Mockey M. Zanello G. Brosson D. Diogon M. Frutos R. (2010). Expression and immunogenicity of the mycobacterial Ag85B/ESAT-6 antigens produced in transgenic plants by elastin-like peptide fusion strategy. *J. Biomed. Biotechnol.* 2010:274346. 10.1155/2010/274346 20414351 PMC2855997

[B12] Gahrtz M. Conrad U. (2009). Immunomodulation of plant function by in vitro selected single-chain Fv intrabodies. *Methods Mol. Biol.* 483 289–312. 10.1007/978-1-59745-407-0_17 19183906

[B13] Helma J. Cardoso M. C. Muyldermans S. Leonhardt H. (2015). Nanobodies and recombinant binders in cell biology. *J. Cell Biol.* 209 633–644. 10.1083/jcb.201409074 26056137 PMC4460151

[B14] Hensel G. Floss D. M. Arcalis E. Sack M. Melnik S. Altmann F. (2015). Transgenic production of an anti hiv antibody in the barley endosperm. *PLOS ONE* 10:e0140476. 10.1371/journal.pone.0140476 26461955 PMC4604167

[B15] Himmelbach A. Zierold U. Hensel G. Riechen J. Douchkov D. Schweizer P. (2007). A set of modular binary vectors for transformation of cereals. *Plant Physiol.* 145 1192–1200. 10.1104/pp.107.111575 17981986 PMC2151723

[B16] Horsch. (1985). A simple and general method for transferring genes into plants. *Science* 227 1229–1231. 10.1126/science.227.4691.1229 17757866

[B17] Ju Shin Y. Kyun Park S. Jung Jung Y. Na Kim Y. Sung Kim K. Kyu Park O. (2015). Nanobody-targeted E3-ubiquitin ligase complex degrades nuclear proteins. *Sci. Rep.* 5:14269. 10.1038/srep14269 26373678 PMC4571616

[B18] Lee D. H. Goldberg A. L. (1998). Proteasome inhibitors: valuable new tools for cell biologists. *Trends Cell Biology* 8 397–403. 10.1016/S0962-8924(98)01346-4 9789328

[B19] Lienert F. Lohmueller J. J. Garg A. Silver P. A. (2014). Synthetic biology in mammalian cells: next generation research tools and therapeutics. *Nat. Rev. Mol. Cell Biol.* 15 95–107. 10.1038/nrm3738 24434884 PMC4032074

[B20] Miroshnichenko S. Tripp J. Nieden U. Neumann D. Conrad U. Manteuffel R. (2005). Immunomodulation of function of small heat shock proteins prevents their assembly into heat stress granules and results in cell death at sublethal temperatures. *Plant J.* 41 269–281. 10.1111/j.1365-313X.2004.02290.x 15634203

[B21] Muyldermans S. (2013). Nanobodies: natural single-domain antibodies. *Annu. Rev. Biochem.* 82 775–797. 10.1146/annurev-biochem-063011-092449 23495938

[B22] Nishimura K. Fukagawa T. Takisawa H. Kakimoto T. Kanemaki M. (2009). An auxin-based degron system for the rapid depletion of proteins in nonplant cells. *Nat. Methods* 6 917–922. 10.1038/nmeth.1401 19915560

[B23] Ravid T. Hochstrasser M. (2008). Diversity of degradation signals in the ubiquitin-proteasome system. *Nat. Rev. Mol. Cell Biol.* 9 679–690. 10.1038/nrm2468 18698327 PMC2606094

[B24] Sakamoto K. M. Kim K. B. Kumagai A. Mercurio F. Crews C. M. Deshaies R. J. (2001). Protacs: chimeric molecules that target proteins to the Skp1-Cullin-F box complex for ubiquitination and degradation. *Proc. Natl. Acad. Sci. U.S.A.* 98 8554–8559. 10.1073/pnas.141230798 11438690 PMC37474

[B25] Sambrook J. (2001). in *Molecular Cloning: A Laboratory Manual*, eds Sambrook J. Russell D. W. (Cold Spring Harbor, NY: Cold Spring Harbor Laboratory).

[B26] Staniek A. Bouwmeester H. Fraser P. D. Kayser O. Martens S. Tissier A. (2013). Natural products – modifying metabolite pathways in plants. *Biotechnol. J.* 8 1159–1171. 10.1002/biot.201300224 24092673

[B27] Tavladoraki P. Benvenuto E. Trinca S. De Martinis D. Cattaneo A. Galeffi P. (1993). Transgenic plants expressing a functional single-chain Fv antibody are specifically protected from virus attack. *Nature* 366 469–472. 10.1038/366469a0 8247156

[B28] ten Hoopen P. Hunger A. Müller A. Hause B. Kramell R. Wasternack C. (2007). Immunomodulation of jasmonate to manipulate the wound response. *J. Exp. Bot.* 58 2525–2535. 10.1093/jxb/erm122 17576762

[B29] Vierstra R. D. (2009). The ubiquitin-26S proteasome system at the nexus of plant biology. *Nat. Rev. Mol. Cell Biol.* 10 385–397. 10.1038/nrm2688 19424292

[B30] Visintin M. Meli G. A. Cannistraci I. Cattaneo A. (2004). Intracellular antibodies for proteomics. *J. Immunol. Methods* 290 135–153. 10.1016/j.jim.2004.04.014 15261577 PMC7126613

[B31] Volkov R. A. Panchuk I. I. Schoffl F. (2003). Heat-stress-dependency and developmental modulation of gene expression: the potential of house-keeping genes as internal standards in mRNA expression profiling using real-time RT-PCR. *J. Exp. Bot.* 54 2343–2349. 10.1093/jxb/erg244 14504302

[B32] Zhao D. Ni W. Feng B. Han T. Petrasek M. G. Ma H. (2003). Members of the Arabidopsis-SKP1-like gene family exhibit a variety of expression patterns and may play diverse roles in Arabidopsis. *Plant Physiol.* 133 203–217. 10.1104/pp.103.024703 12970487 PMC196598

[B33] Zhou P. (2005). Targeted protein degradation. *Curr. Opin. Chem. Biol.* 9 51–55. 10.1016/j.cbpa.2004.10.012 15701453

[B34] Zhou P. Bogacki R. McReynolds L. Howley P. M. (2000). Harnessing the ubiquitination machinery to target the degradation of specific cellular proteins. *Mol. Cell* 6 751–756. 10.1016/S1097-2765(00)00074-5 11030355

