# Supplementary material for: Nanobody-Directed Specific Degradation of Proteins by the 26S-Proteasome in Plants
Source: Front Plant Sci. 2018 Feb 9;9:130. doi: 10.3389/fpls.2018.00130 (PMC5811635; doi:10.3389/fpls.2018.00130)
Supplement: Supplementary file 1 [file Data_Sheet_1.docx]

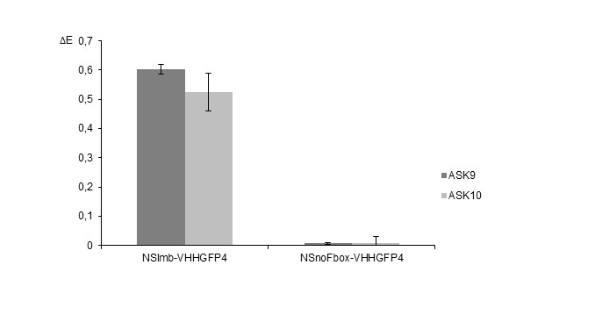


Suppl. Figure: Selective binding of NSlmb-VHHGFP4 to ASK proteins *in vitro*. ASK9 and ASK10 proteins were adsorbed to wells of microtiter plates. NSlmb-VHHGFP4 and NSnoFbox-VHHGFP4 were applied to the microtiter plates in relevant and equal concentrations (0.01 µg/100 µL for NSlmb-VHHGFP4 and NSnoFbox-VHHGFP4), and the binding of F-box derivatives to either ASK9 or ASK10 was measured using the cmyc tag. ΔE: Extinction difference. ASK9, ASK10: S-phase kinase-associated proteins from *Arabidopsis* (Takahashi et al., 2004). ASK9 and ASK10 sequences (kindly provided by Marcel Quint, Universität Halle/Saale, Germany) were amplified using the primer sequences described to add suitable restriction sites (*Xho*I and *Bam*HI). Fragments were cloned into the expression vector pET23a (Novagen) via TOPO cloning vector (Thermo Fisher Scientific). The proteins were produced in E. coli and purified via IMAC (immobilized metal ion affinity chromatography). Proteins were extracted in 50 mM Tris buffer (pH 8.0). The extracts were centrifuged (75 600 x g, 30 min, 4°C) and mixed with Ni-NTA agarose resin (Protino, Macherey-Nagel) that had previously been washed with water twice. After mixing overnight at 4°C, the mixture was applied to a chromatography column. Thereafter, the column was extensively washed (50 mM NaH2PO4, 300 mM NaCl, 30 mM imidazole, pH 8.0). Recombinant proteins were then eluted from the column with elution buffer (50 mM NaH2PO4, 300 mM NaCl, 250 mM Imidazole, pH 8.0), placed in dialysis bags, concentrated in PEG 6000 and dialyzed against PBS. The resulting proteins were diluted in Phage PBS (100 mM NaCl, 32 mM NaH2PO4, 17 mM NaH2PO4, pH 7.2) at a concentration of 0.3 µg/100 µL and placed in Immunoplate MaxiSorp wells (Nalge Nunc International). After incubation overnight at room temperature, the wells were saturated with 3% BSA in PBS-T (Gahrtz and Conrad, 2009). NSlmb-VHHGFP4 or NSnoFbox-VHHGFP4 were produced in plants and purified by affinity chromatography using the cmyc tag. F-box derivatives were diluted in 1% BSA in PBS-T to concentrations of 0.01 µg/100 µL for NSlmb-VHHGFP4 and NSnoFbox-VHHGFP4, applied to the wells (100 µL each) and incubated for 1 h at 25°C. After extensive washing with PBS-T, anti-cmyc antibodies diluted in 1% BSA in PBS-T were added for 1 h at 25°C. After extensive washing, rabbit anti-mouse IgG (alkaline-phosphatase-linked whole antibody, SIGMA, diluted 1:2000 in 1% BSA and PBS-T), was applied for 1 h at 25°C. After further washing the enzymatic substrate, p-nitrophenyl phosphate (pNPP) in 0.1 M diethanolamine-HCl (pH 9.8) was added, and the absorbance signal was measured at 405 nm after 1 h incubation at 37°C. Measured values from control experiments performed in parallel (same handling procedure but without the antigen incubation step) were subtracted.

References

Gahrtz, M., and Conrad, U. (2009). Immunomodulation of plant function by in vitro selected single-chain Fv intrabodies. Methods in molecular biology (Clifton, N.J.) *483*, 289-312.

Takahashi, N., Kuroda, H., Kuromori, T., Hirayama, T., Seki, M., Shinozaki, K., Shimada, H., and Matsui, M. (2004). Expression and interaction analysis of Arabidopsis Skp1-related genes. Plant & cell physiology *45*, 83-91.
